# Effect of boiling and roasting on the physicochemical properties of Djansang seeds (*Ricinodendron heudelotii*)

**DOI:** 10.1002/fsn3.1163

**Published:** 2019-09-26

**Authors:** Emmanuel Edie Kinge, Fabrice Tonfack Djikeng, Mallampalli Sri Lakshmi Karuna, François Zambou Ngoufack, Hilaire Macaire Womeni

**Affiliations:** ^1^ School of Agriculture and Natural Resources Catholic University Institute of Buea Buea Cameroon; ^2^ Department of Biochemistry, Faculty of Science University of Dschang Dschang Cameroon; ^3^ CSIR‐Indian Institute of Chemical Technology, Centre for Lipid Research Tarnaka Hyderabad India

**Keywords:** antioxidant activity, boiling, Djansang, lipid quality, nutritional composition, roasting

## Abstract

This study was aimed at determining the effect of boiling and roasting on physicochemical properties of Djansang seeds. Dried Djansang seeds were divided into three groups: one group was boiled for 5, 10, and 15 min; another group was traditionally roasted for 5, 10, and 15 min, and the last group was left unprocessed and served as the control. Polyphenols were extracted from the processed seeds using the maceration method, and their content and antioxidant activity were evaluated. Oils were extracted from the dried seeds by Maceration method, and the quality was analyzed by determining their peroxide value (PV), thiobarbituric acid value (TBA), iodine value (IV), and acid value (AV). The changes in proximate composition and mineral content of the processed samples were also evaluated using standard methods. Results showed that traditional roasting significantly decreases (*p* < .05) the polyphenol content and antioxidant activity of Djansang seeds as compared to boiling. The analysis of oil showed that traditional roasting and boiling significantly reduce the quality of Djansang seed oil (PV: 10.85–38.49 meq O_2_/kg; TBA: 1.80–3.20 ppm; AV: 0.28%–0.82%; and IV: 104.27–98.11 g I_2_/g for roasted samples, and PV: 10.85–27.52meq O_2_/kg; TBA: 1.45–2.28 ppm; AV: 0.56%–0.96%; and IV: 105.87–102.96 g I_2_/g for boiled samples) compared to the control (PV: 9.96 meq O_2_/kg; TBA: 1.01 ppm; AV: 0.11%; and IV: 104.83 g I_2_/g) and that traditionally roasted samples were the most affected. The proximate and mineral composition of Djansang was also affected during processing. Boiling for 5 and 10 min (*BNS *5 min and 10 min) and traditional roasting for 5 min (*TRNS* 5 min) appear to be the best processing methods of Djansang for production of Djansang‐based foods like Djansang sauce.

## 1 INTRODUCTION

*Ricinodendron heudelotii* (Baill.) is a fast‐growing tree, which produces edible seeds, traditionally used in many countries of Africa in the preparation of foods. It is called the Djansang tree and belongs to the family Euphorbiaceae (Tchoundjeu & Atangana, 2006). The proximate composition of Djansang shows that the seeds are rich in proteins (~22%), lipid (~46%), carbohydrates (~25%), and mineral elements. They also contain nonnegligible amount of phenolic compounds (~5 mg GAE/g) and have been shown to have good antioxidant and antibacterial properties (Ene‐obong, Onuaha, Aburime, & Mbah, 2018; Oyono et al., 2014). The seeds of *Ricinodendron heudelotii* are rich in polyunsaturated fatty acids (~79%) among which the most represented essential fatty acid is linoleic acid (~28.3%). They also contain several essential amino acids (Tchoundjeu & Atangana, 2006). Lysine is usually limiting in many vegetable foods but was found present in amount exceeding the reference protein requirement (FAO/WHO, 2011) in *Ricinodendron heudelotii* defatted flour (Mezajoug, Arab‐Tehrany, Tchiégang, & Linder, 2011).

Djansang seeds are generally sun dried or smoked. However, sun drying is the best processing technique, as it improves the color of the seeds and increases their acceptability. Consumers prefer Djansang with light yellow color. During drying, it is important to avoid that it rains on the seeds, because they become kaki or black in color. Djansang is generally used in the preparation of food in Africa as spice or principal ingredient. It is used in the preparation of Djansang sauce, which is highly appreciated by the population of the South‐West region of Cameroon. The seeds are generally cooked by boiling and roasting.

It is well known that during thermal processing, the heat can have a detrimental effect on the nutritional and functional properties of foods (Fokwen et al., 2019; Kanu, Kalu, & Okorie, 2015). Then, the thermal treatment of foods can lead to chemical changes that can affect its phenolic content, antioxidant property, lipid quality, proximate composition, and mineral content. For example, these treatments can facilitate the oxidation of lipids and nonenzymatic browning reactions, which can negatively affect the nutritional quality of foods by decreasing it, causing a significant decrease in essential fatty acids, essential amino acids, and carbohydrates. The concentration of vitamins can also be reduced as well as the protein digestibility (Cuvelier & Maillard, 2012) of the food. Additionally, these chemical alteration reactions may generate toxic compounds in edible seeds and the derived products, which can be harmful for the consumers (Djikeng et al., 2017). The heat labile phenolic compounds present in the food can also lost, leading to a decrease in their antioxidant and antimicrobial properties, thus reducing their health benefits.

Many studies have reported the effect of processing temperature and time on the phenolic compounds, antioxidant activity, lipid quality, and nutritional value of foods. Some studies on nuts include the following: the effect of boiling and roasting on the lipid quality, proximate composition, and mineral content of African walnut seeds (Djikeng et al., 2017); the effects of cooking methods and temperatures on nutritional and quality characteristics of anchovy (*Engraulis encrasicholus*) (Uran & Gokoglu, 2014); and the effect of cooking methods on total phenolics and antioxidant activity of selected green vegetables (Turkmen, Sari, & Velioglu, 2005). Though considerable reports are available on the effects of processing on the phenolic content, antioxidant activity, lipid quality, and nutritional value of foods, there is however very limited reports on the effects of boiling and roasting on the phenolic content, antioxidant activity, lipid quality, and nutritional composition of Djansang. We can hypothesize that boiling and roasting times significantly affect the phenolic content, antioxidant activity, lipid quality, proximate composition, and mineral content of *Ricinodendron heudelotii* seeds.

Therefore, the aim of this study is to evaluate the effect of boiling and roasting time on the phenolic content, antioxidant activity, lipid oxidation, proximate composition, and mineral content of Djansang seeds.

## 2 MATERIAL AND METHODS

### 2.1 Material

Sun‐dried Djansang seeds (*Ricinodendron heudelotii)* were purchased from Muea local market, Buea, South‐West Region, Cameroon, in December 2017. All chemicals and reagents used were of analytical reagent grade.

### 2.2 Methods

#### 2.2.1 Sample preparation and processing

Djansang seeds were divided into three different groups (G1, G2, and G3). The first group was now divided into three different subgroups of 300 g each, SG1, SG2, and SG3, which were traditionally roasted in cooking pot by continuous stirring for 5, 10, and 15 min respectively, and were affected the codes TRNS 5min, TRNS 10 min, and TRNS 15min, respectively. The temperature between the heat source and cooking pot was ranged between 200 and 220°C.

The second group was also divided into three subgroups of 300 g each, SG1', SG2', and SG3' which were boiled at about 98°C for 5, 10, and 15 min, respectively, and were affected the codes BNS 5 min, BNS 10 min, and BNS 15 min, respectively.

The last group (group 3) remained unprocessed and served as control and were affected the codes control.

After this, all the above‐mentioned samples were dried in an electric oven for 48 hr at 50°C before being used for further analysis.

#### 2.2.2 Extraction of Djansang polyphenols

Dried Djansang samples were grounded to 1 mm diameter using a grinding machine (Moulinex). 50 g of each sample powder *(Control, TRNS *5 min,* TRNS *10 min,* TRNS *15 min,* BNS *5 min,* BNS 10* min, and *BNS* 15 min*)* was extracted with 400 ml of methanol for 48 hr at room temperature. The mixture was regularly subjected to shaking during the extraction. The extract was filtered with a Whatman No. 1 filter paper, and residue was again extracted with 200 ml of methanol to ensure maximum extraction of phenolic compounds. The combined filtrates were subjected to rotary evaporation at 40°C under reduced pressure for the removal of the solvent. The dried extract was used for the determination of the total phenolic content and antioxidant activities.

#### 2.2.3 Total polyphenol of determination

The total phenolic content of Djansang seeds was determined using the Folin–Ciocalteu colorimetric method, as described by Gao, Ohlander, Jeppsson, Björk, and Trajkovski (2000). In a test tube of 5 ml volume, 20 μl of a 2 mg/ml extract solution was added, followed by the Folin–Ciocalteu reagent (0.2 ml) and distilled water (2 ml). After 3 min incubation of the solution mixture at room temperature, 1 ml of 20% sodium carbonate solution was added and the mixture re‐incubated for 20 min under the same conditions. The absorbance of the resulting blue colored solution was measured at 765 nm using a spectrophotometer. The total phenolic content of the extract was calculated from the gallic acid standard curve and expressed as milligrams equivalents gallic acid per gram of extract.

#### 2.2.4 Antioxidant activity of Djansang seeds

The ability of each extract to scavenge the DPPH radical was determined according to the method of Braca, Sortino, Politi, Morelli, and Mendez (2002). A total of 4.5 ml of 0.002% alcoholic solution of DPPH was added to 0.5 ml of different concentrations (250, 500, 1,000, and 2,000 μg/ml) of samples and standard solutions separately, in order to have final concentrations of products of 25–200 μg/ml. The samples were kept at room temperature in the dark and after 30 min, and the absorbance of the resulting solution was measured at 517 nm. The absorbance of the samples, control, and blank was measured in comparison with methanol. The antioxidant activity (AA) was calculated according to the formula:

$$\text{AA}\%=\left[\frac{({\text{Abs}}_{\text{control}}-{\text{Abs}}_{\text{sample}})\times 100}{{\text{Abs}}_{\text{control}}}\right]$$

#### 2.2.5 Quality of Djansang seeds oils

The determination of the peroxide value of Djansang seed samples was made following the spectrophotometrical IDF standard method, 74A: 1991. Its iodine and acid values were determined according to the procedure of AOCS (2003) Official Method CD 1‐25 (2003) and CD 3d‐63 (2003), respectively. Finally, its thiobarbituric acid value was evaluated as described by Draper and Hadley (1990).

#### 2.2.6 Proximate composition of Djansang seeds

Moisture, fat, ash, and protein content of all the samples were determined using standard analytical methods described by AOAC (1990) procedures. Moisture content was determined by drying Djansang seeds in oven at 103°C until a constant weight was achieved according to the AOAC procedures 925.40. Ash content was determined by incineration of Djansang seeds at 550°C according to the AOAC procedures 942.05. Nitrogen (N) content was determined using micro‐Kjeldahl method, according to AOAC procedures 984.13, and the protein content was calculated as N × 6.25. Lipid content was determined using Soxhlet apparatus with hexane, following AOAC 963.15 methodology. The total percentage carbohydrate content was determined by the difference method as reported by Onyeike, Anyalogbu, and Monanu (2015). This method involved adding the total values of crude protein, crude fat, moisture, and ash constituents of the sample and subtracting it from 100. All samples were analyzed in triplicate.

#### 2.2.7 Mineral content of Djansang seeds

For the determination of minerals, Djansang seeds were ashed at 550°C and dissolved with 10 ml of 20% HCl in a beaker and then filtered into a 100‐ml standard flask to determine the mineral content. Calcium (Ca), magnesium (Mg), sodium (Na), potassium (K), and iron (Fe) were determined by atomic absorption spectrometer (Varian 220FS Spectra AA). Phosphorus (P) was determined colorimetrically using the vanadomolybdate, according to AOAC procedure 965.17 (1999). Mineral contents of the samples were determined from calibration curves of standards minerals. All samples were analyzed in triplicate.

### 2.3 Statistical analysis

Results obtained in the present study were subjected to one‐way analysis of variance (ANOVA) with Student–Newman–Keuls tests using Graphpad‐InStat version 3.05, to evaluate the statistical significance of the data. A probability value at *p* < .05 was considered statistically significant.

## 3 RESULTS AND DISCUSSION

### 3.1 Effect of processing on the total phenolic content (TPC) of Djansang seeds

**Figure 1:**
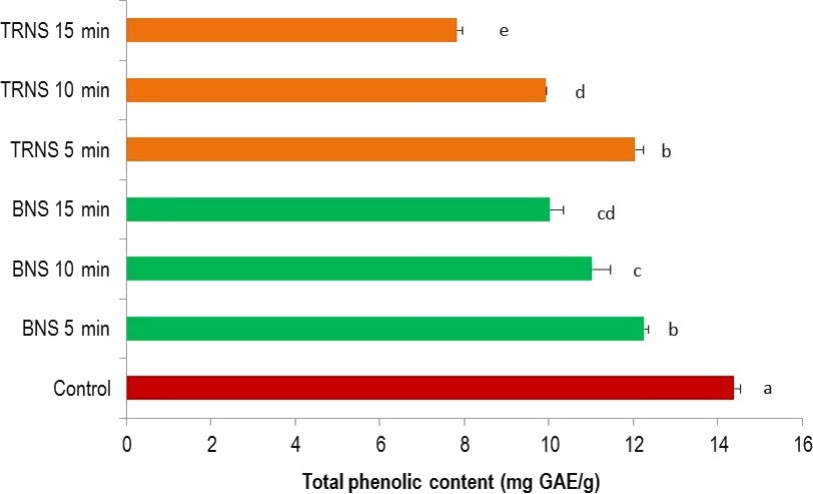
Changes in total phenolic content of Djansang during processing. Where CNS, raw Djansang seeds (control); BNS 5 min, boiled Djansang seeds 5 minutes; BNS 10 min, boiled Djansang seeds 10 minutes; BNS 15 min, boiled Djansang seeds 15 minutes; TRNS 5 min, traditional roasting Djansang seeds 5 minutes; TRNS 10 min, traditionally roasted Djansang seeds 10 minutes; TRNS 15 min, traditionally roasted Djansang seeds 15 minutes

The changes in total phenolic content of processed Djansang seed samples compared to the control (CNS) are presented in Figure 1. A significant decrease (*p* < .05) in TPC was registered in all the processed samples compared to the control. The TPC was significantly decreasing (*p* < .05) with processing time. The lowest value was registered with TRNS 15 min. Generally, the phenolic content of boiled samples was higher than those of roasted ones. The fact that the total phenolic content of plant materials decreases with the processing time has already been reported. Djikeng et al. (2018) showed that the TPC of fermented cocoa beans was significantly decreasing (*p* < .05) with the roasting period. The higher TPC obtained with boiled samples compared to roasted ones might be attributed to its low processing temperature, which was 98°C while that of roasting was ranged between 200 and 220°C. The significant decrease in TPC in all the processed samples can be attributed to the volatilization of low molecular weight phenolic compounds present as well as heat‐induced polymerization. These results are in accordance with those of Endraiyani (2011) who demonstrated that the total polyphenols of cocoa pulps were significantly decreasing with the number of pasteurization. In the same line, Rizki, Kzaiber, Elharfi, Ennahli, and Hanine (2015) reported that the TPC of sesame seeds significantly decreases with roasting time.

### 3.2 Effect of processing on the antioxidant activity of Djansang seeds

**Figure 2:**
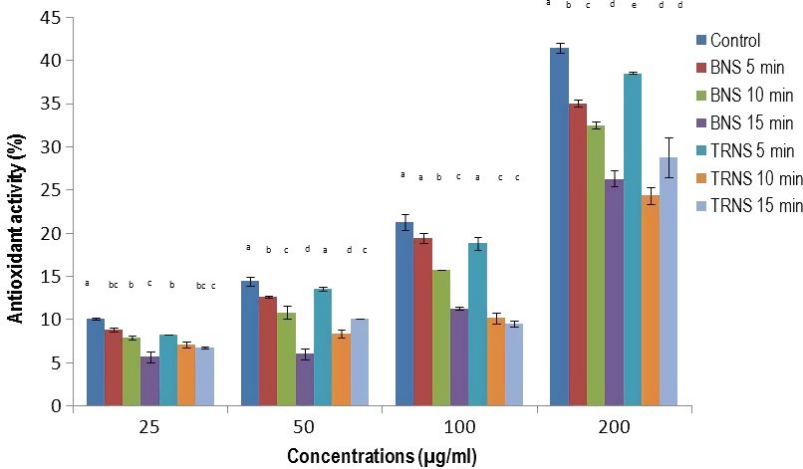
Changes in radical scavenging activity of Djansang seeds during processing. Where CNS, raw Djansang seeds (control); BNS 5 min, boiled Djansang seeds 5 minutes; BNS 10 min, boiled Djansang seeds 10 minutes; BNS 15 min, boiled Djansang seeds 15 minutes; TRNS 5 min, traditional roasting Djansang seeds 5 minutes; TRNS 10 min, traditionally roasted Djansang seeds 10 minutes; TRNS 15 min, traditionally roasted Djansang seeds 15 minutes

The effect of different processing conditions and time on the antioxidant activity of Djansang is illustrated in Figure 2. Globally, the activity of the extract was significantly increasing (*p* < .05) with their concentrations. The unprocessed Djansang samples have exhibited the highest antioxidant activity, and this at all concentration. However, compared to it, the activities of the processed samples were significantly lower (*p* < .05). As previously observed with the total phenolic content, the roasted samples showed the lowest antioxidant activity. In previous studies, the relation between TPC and antioxidant activity of plant extracts was reported. Authors showed that plant extracts having high concentration in phenolic compounds were also exhibiting the highest antioxidant activity (Bouba, Njintang, Scher, & Mbofung, 2010; Djikeng et al., 2017; Womeni et al., 2016; Womeni, Tonfack, Tiencheu, & Linder, 2013). From these results, it appears clear that roasting significantly reduced both polyphenols and antioxidant activity of Djansang seeds. These results are in line with those reported by Djikeng et al. (2018) which showed that roasting significantly decreased the antioxidant activity of fermented cocoa beans.

### 3.3 Changes in the quality of Djansang seed oil during processing

#### 3.3.1 Peroxide value (PV)

The primary oxidation state of oils and fats is generally evaluated by determining their peroxide value which is a chemical test of characterization of hydro peroxides, the main primary oxidation products in lipids (Djikeng et al., 2017; Iqbal & Bhanger, 2007). The changes in peroxide value of Djansang oil samples are presented in Table 1. Apart from the sample BNS 5 min that have shown similar peroxide value with the control, all the other samples have exhibited significantly higher (*p* < .05) peroxide values. Oil samples extracted from roasted Djansang seeds have exhibited peroxide values varying from 12.55 to 38.49 meq O_2_/kg, while the peroxide value of oils obtained from the boiled seeds varied between 10.85 and 27.52 meq O_2_/kg. Generally, the peroxide value obtained from boiled samples was significantly lower than those obtained from roasted samples. However, the peroxide value of all the processed samples was significantly higher than 10 meq O_2_/kg, which is the highest peroxide value to be obtained in good quality oil as recommended by FAO/WHO (2009). The increase in peroxide value observed in all the processed samples can be attributed to the formation of hydro peroxides. The fact that the peroxide values of oil samples obtained after roasting is higher than those obtained after boiling indicates the higher formation rate of hydro peroxides in them. The differences in processing temperatures can explain the observed results. It has been demonstrated that heat facilitates the initiation of lipid oxidation which leads to the formation of free radicals and which in turn reacts with molecular oxygen and form hydro peroxides at the propagation stage (Anwar, Qayyum, Hussian, & Iqbal, 2010). These results are in accordance with those reported by Tenyang et al. (2017) and Djikeng et al. (2018) who, respectively, show that the peroxide values of oils extracted from sesame seeds and fermented cocoa beans were increasing with the roasting temperature and time.

**Table 1:** Changes in peroxide, TBA, acid, and iodine vales of Djansang seed oil during processing

	Peroxide value (meq O_2_/kg)	TBA value (ppm)	Acid value (% Oleic acid)	Iodine value (g I_2_/100 g)
Control	9.96 ± 0.95^a^	1.01 ± 0.07^a^	0.11 ± 0.01^d^	104.83 ± 1.15^ac^
TRNS 5 min	12.55 ± 0.19^b^	1.80 ± 0.00^b^	0.28 ± 0.00^c^	104.27 ± 0.62^a^
TRNS 10 min	25.72 ± 0.00^c^	3.29 ± 0.02^c^	0.55 ± 0.00^b^	101.56 ± 0.27^b^
TRNS 15 min	38.49 ± 1.25^d^	3.20 ± 0.05^c^	0.82 ± 0.02^b^	98.11 ± 0.14^c^
BNS 5 min	10.85 ± 0.00^a^	1.45 ± 0.06^d^	0.56 ± 0.00^b^	105.87 ± 0.22^c^
BNS 10 min	19.83 ± 1.94^e^	1.67 ± 0.04^e^	0.84 ± 0.03^a^	105.13 ± 0.17^c^
BNS 15 min	27.52 ± 0.84^f^	2.28 ± 0.00^f^	0.96 ± 0.07^a^	102.96 ± 0.31^b^

Note

Data are presented as mean ± *SD* (*n* = 3). Superscript letters (a‐g) Means within each column with different superscripts are significantly (*p* < .05) different.

Abbreviations: BNS 10 min, boiled Djansang seeds 10 minutes; BNS 15 min, boiled Djansang seeds 15 minutes; BNS 5 min, boiled Djansang seeds 5 minutes; CNS, raw Djansang seeds (control); TRNS 10 min, traditionally roasted Djansang seeds 10 minutes; TRNS 15 min, traditionally roasted Djansang seeds 15 minutes; TRNS 5 min, traditional roasting Djansang seeds 5 minutes.

#### 3.3.2 Thiobarbituric acid value (TBA value)

This test is generally used to evaluate the secondary oxidation state of oils and fats. It gives an idea of the concentration of malondialdehyde present, which is a product obtained from the secondary oxidation of polyunsaturated lipids (Iqbal & Bhanger, 2007). The changes in TBA values of Djansang oil samples during processing of their seeds are presented in Table 1. A significant increase (*p* < .05) in TBA value was registered in all the processed samples compared to the control. As previously observed with the peroxide value, oils obtained from roasted Djansang seeds exhibited significantly higher (*p* < .05) TBA values compared to those extracted from boiled seeds. This is the proof that the amount of malondialdehyde significantly increases (*p* < .05) with roasting than boiling. The difference in processing temperature of these samples can be attributed to the temperature used. The roasting temperature might be acting as a good catalyst of the formation of hydro peroxides and to their decomposition into secondary oxidation products among which malondialdehyde. These results are in line with those of Djikeng et al. (2017) who show that roasting was significantly increasing the TBA value of African walnut than boiling.

#### 3.3.3 Acid value

The determination of the acid value of oils and fats generally informs on the hydrolysis of their triglycerides that leads to the release of free fatty acids which are the main substrates of lipid oxidation reactions. These reactions are catalyzed by the moisture, high temperatures, and enzymes such as lipase (Tenyang et al., 2017). The variations in acidity of oil samples during processing of Djansang seeds are reported in Table 1. A significant increase (*p* < .01) in acid value was recorded in all the processed samples compared to the control. However, oil samples obtained from boiled Djansang seeds exhibited significantly higher acid values compared to those extracted from roasted samples. This can be attributed to the hydrolytic action of both water and temperature used during the process. The acid value in all samples was lower than 4% which is the maximum recommended acid value in crude oils (FAO/WHO, 2009). These results are in accordance with those reported by Djikeng et al. (2017) and Tenyang et al. (2017) who demonstrated that the acid value of oil samples significantly increases with the processing time and temperature.

#### 3.3.4 Iodine value

This parameter generally gives an idea on the degree of unsaturation of oils and fats. During lipid oxidation, the fatty acid double bonds are attacked by free radicals. This results in the decrease of their number of unsaturation (Tynek, Hazuka, Pawlowicz, & Dudek, 2001). The changes in iodine value of Djansang oil samples during processing of their seeds are presented in Table 1. Globally, the iodine value was significantly decreasing (*p* < .05) with the processing time. The highest decrement rate was registered with oil samples obtained from roasted seeds. The iodine value of oil samples was significantly decreasing with processing time. The lowest iodine value of oil samples obtained from roasted seeds compared to the boiled ones can be attributed to its high processing temperature that has facilitated the attack of the fatty acid double bonds by the free radicals. The iodine values obtained in this study were ranged between 98.11 and 105.87 g I_2_/100 g, which were not far from those reported by Nzali, Tchiengang, Sandjon, and Meurens (2016) who showed that the iodine value of *Ricinodendron heudelotii* varies between 103.46 and 105.38 g I_2_/100 g. These results are in line with those reported by Djikeng et al. (2017) who demonstrated that roasting and boiling temperature and time were significantly reducing the iodine value of African walnut oil.

### 3.4 Effect of processing on the proximate composition of Djansang seeds

The variations in proximate composition of Djansang seeds are reported in Table 2. It appears that the amount of ash varied from 7.00% to 10.00%. A similar range was reported in the seeds of this same plant by Coulibaly, Adam, et al. (2018)) and Coulibaly, N'dri, et al. (2018). No significant difference was observed between the ash content of control and that of TRNS 15 min. However, the total ash content of all the other samples was significantly higher (*p* < .05) compared to those mentioned above. These suggest that almost all the processing methods used increase the ash content of Djansang seeds. These results are in accordance with those reported by Djikeng et al. (2017) and Amaiz, Carmen, Gutierrez, Perez, and Alvarez (2012) who respectively demonstrated that boiling and roasting significantly increased the ash content of African walnuts and cocoa beans.

**Table 2:** Proximate composition (dry basis) of raw and processed Djansang seeds

	Protein (%)	Lipid (%)	Ash (%)	Carbohydrate (%)
Control	37.20 ± 0.33^a^	46.97 ± 1.70^ab^	7.00 ± 0.11^a^	8.83 ± 0.15^a^
TRNS 5 min	36.55 ± 0.12^ab^	46.18 ± 0.33^b^	9.00 ± 0.20^b^	8.27 ± 0.22^a^
TRNS 10 min	34.60 ± 0.21^b^	46.84 ± 0.35^ab^	10.00 ± 0.12^b^	8.56 ± 0.14^a^
TRNS 15 min	35.15 ± 0.25^b^	50.74 ± 2.11^a^	8.00 ± 0.08^a^	6.11 ± 0.14^bc^
BNS 5 min	35.56 ± 1.12^ab^	46.57 ± 0.20^ab^	10.00 ± 1.34^b^	7.87 ± 0.22^ac^
BNS 10 min	36.25 ± 0.31^ab^	46.62 ± 0.22^ab^	9.00 ± 0.30^b^	8.13 ± 0.17^a^
BNS 15 min	34.12 ± 0.47^c^	47.92 ± 1.11^ab^	10.00 ± 0.27^b^	7.96 ± 0.31^ac^

Note

Data are presented as mean ± *SD* (*n* = 3). Superscript letters (a‐g) Means within each column with different superscripts are significantly (*p* < .05) different.

Abbreviations: BNS 10 min, boiled Djansang seeds 10 minutes; BNS 15min, boiled Djansang seeds 15 minutes; BNS 5 min, boiled Djansang seeds 5 minutes; CNS, raw Djansang seeds (control); TRNS 10 min, traditionally roasted Djansang seeds 10 minutes; TRNS 15 min, traditionally roasted Djansang seeds 15 minutes; TRNS 5 min, traditional roasting Djansang seeds 5 minutes.

The analysis of macronutrients showed that Djansang is rich in proteins (34.12% to 37.20%), lipids (46.57% to 50.74%), and carbohydrates (6.11% to 8.83%). The amount of lipid obtained in this study was close to those reported by Nzali et al. (2016) and Ezekwe, Besong, and Johnson (2014) who respectively obtained 51.03% and 44.70% as lipid content of *Ricinodendron heudelotii*. However, the amount of protein obtained in this study was significantly higher than that reported by Ezekwe et al. (2014) who obtained a total protein content of 31.4% of the same seeds. However, these value falls in the range of 24.3% to 65.2% as reported by Tsware and Usman (1998) with the same seeds. Concerning the carbohydrate content, the value obtained in this study was close to those reported by Coulibaly, Adam, et al. (2018 and Coulibaly, N'dri, et al. (2018 who obtained a total carbohydrate content varying between 5.60% and 13.10%. Generally, the amount of proteins and carbohydrates was significantly decreasing with the treatments. This can be explained by the fact that these substrates are involved in nonenzymatic browning reactions. Similar results were previously reported by Ajala and Ojewande (2014), Ihemeje, Ukauwa, and Ekwe (2015), and Djikeng et al. (2017). Globally, no significant difference was registered between the lipid content of the control and those of the processed seeds.

The changes in proximate composition of Djansang seeds during processing are presented in Table 2. From the same table (Table 2), we can notice that the amount of ash varied from 7% to 10% which was not in accordance with (Tchiégang, Mezajoug Kenfack and Kapsue 2000; Manga, Fondoun, Kengue, & Tchiegang, 2000; Kapseu, 1998; Dandjouma, Tchiegang, Ndjouenkeu, Kapseu, and Mbofing, 2000; Ekam 2003; Tane, 1997; Tsware & Usman, 1998). No significant difference was observed between the ash content of the control (CNS) and TRNS 15 min. However, the total ash content of TRNS 5 min, TRNS 10 min, BNS 5 min, BNS 10 min, and BNS 15 min was significantly higher (*p* < .05) compared to those mentioned above. This suggests that traditional roasting and boiling increase the amount of ash in Djansang seeds. These results are in agreement with those of Nwafor, Egonu, Nweze, and Ohabueny (2017) who demonstrated that the ash content of Fabaceae seeds increased when processed. The analysis of the macronutrients composition showed that Djansang seeds are rich in lipids (46.18%–50.74%), proteins (34.12%–37.20%), and carbohydrates (6.11%–8.83%). The amount of lipids was similar to that reported by Manga et al. (2000) who showed that the lipid content of Djansang seeds varied between 45% and 67%. Those of proteins and carbohydrates were close to the data reported by Tane (1997) who demonstrated that the protein and carbohydrate contents of Djansang seeds fall in the ranges of 24%–65% and 5.6%–9.3%, respectively.

Generally, the amount of lipids for almost all the samples was not significantly affected (*p* < .05) with the treatments. However, there was a significant increase for TRNS 15 min, and this might be due to the loss of water which facilitates lipid extraction. For proteins, there was generally a significant reduction (*p* < .05) for both traditionally roasted and boiled samples, with the reduction increasing with increase in processing time. This reduction can be attributed to the Maillard reaction, as proteins are substrates of nonenzymatic browning (Tenyang et al., 2017).

Concerning the total carbohydrates content, there was no significant difference (*p* < .05) between the control (CNS) and samples of TRNS 5 min, TRNS 10 min, and BNS 10 min; however, there was significant reduction (*p* < .05) between TRNS 15 min, BNS 5 min, and BNS 15 min as compared to the control. These results are in agreement with those of Ihemeje et al. (2015) who demonstrated that the amount of carbohydrate of walnut seeds significantly decreases (*p* < .05) with the treatments.

### 3.5 Changes in the mineral content of Djansang seeds during processing

The changes in mineral composition of Djansang seeds during processing are presented in Table 3. Result showed that Djansang seeds contained several mineral elements which are recognized to play very important functions in the human body such as energy production, transmission of nerve impulses, enzymatic reactions, and other biological reactions (Steinberg, Bearden, & Keen, 2003). From the Table 3, it is clear that the amount of iron significantly (*p* < .05) increased with the treatment compared to the control. The increase in iron observed with the processed samples can be attributed to the fact that these treatments increase the digestibility of Djansang seeds and then initiate the release and increase of iron. Generally, the amount of iron in all samples was ranged between 20.56 and 71.39 mg/100 g. These values were significantly higher (*p* < .05) than those reported by Coulibaly, Adam, et al. (2018 and Coulibaly, N'dri, et al. (2018 who reported that the iron content of *Ricinodendron heudelotii* kernel flour contained 0.00% of iron. However, the iron content obtained in this study was significantly higher (*p* < .05) than that reported Ene‐Obong et al. (2018) who showed that Djansang seeds consumed in Nigeria, contained 15.28 mg/100 g. The presence of iron in these seeds is very important, for humans and animals, since this mineral is an important part of the respiratory pigment, myoglobin, hemoglobin, and several enzymes. Its deficiency leads to anemia which is a severe nutritional diseases in the world nowadays Loumouamou, Silou, and Desobry (2010).

**Table 3:** Changes in mineral content of Djansang seeds during processing

	P (mg/100 g)	K (mg/100 g)	Na (mg/100 g)	Fe (mg/100 g)	Ca (mg/100 g)	Mg (mg/100 g)
Control	15.40 ± 0.11^e^	1,107.20 ± 11.31^b^	53.80 ± 6.02^ac^	20.56 ± 0.41^b^	856.80 ± 11.12^b^	189.50 ± 3.08^a^
TRNS 5 min	60.39 ± 0.23^a^	2,965.20 ± 8.26^a^	119.00 ± 2.48^b^	71.39 ± 0.90^a^	2,565.20 ± 10.87^a^	764.50 ± 3.43^b^
TRNS 10 min	21.74 ± 0.34^d^	938.20 ± 5.21^c^	46.40 ± 5.51^a^	49.50 ± 0.23^c^	1,765.00 ± 4.55^c^	498.50 ± 7.71^c^
TRNS 15 min	18.57 ± 0.22^c^	1,289.20 ± 0.76^d^	61.70 ± 1.07^c^	53.39 ± 0.94^d^	1,176.00 ± 2.13^d^	228.40 ± 3.64^d^
BNS 5 min	19.84 ± 0.19^c^	898.00 ± 3.45^e^	46.40 ± 0.87^a^	46.58 ± 0.13^e^	1,546.90 ± 12.34^e^	423.50 ± 1.90^e^
BNS 10 min	19.84 ± 0.88^c^	918.00 ± 12.45^c^	46.40 ± 5.44^a^	54.60 ± 0.66^d^	2,032.00 ± 22.69^f^	658.90 ± 8.74^f^
BNS 15 min	54.05 ± 0.58^b^	435.60 ± 4.91^f^	26.70 ± 4.62^d^	59.71 ± 0.81^f^	2,478.00 ± 8.65^g^	756.90 ± 7.61^b^

Note

Data are presented as mean ± *SD* (*n* = 3). Superscript letters (a‐g) Means within each column with different superscripts are significantly (*p* < .05) different.

Abbreviations: BNS 10 min, boiled Djansang seeds 10 minutes; BNS 15 min, boiled Djansang seeds 15 minutes; BNS 5 min, boiled Djansang seeds 5 minutes; CNS, raw Djansang seeds (control); TRNS 10 min, traditionally roasted Djansang seeds 10 minutes; TRNS 15 min, traditionally roasted Djansang seeds 15 minutes; TRNS 5 min, traditional roasting Djansang seeds 5 minutes.

Concerning the calcium content, it also significantly increased with the treatment. The highest rate of increment was observed in boiled samples. Globally, the calcium content of all the samples was ranged between 856 and 2,565 mg/100 g which was significantly higher than 627 mg/100 g as reported by Ene‐Obong et al. (2018) with the same seeds. However, the amount of calcium obtained in this study was significantly higher than that reported by Coulibaly, Adam, et al. (2018 and Coulibaly, N'dri, et al. (2018, who demonstrated that the calcium content of Djansang kernel flour ranged between 1.4% and 1.8%. Djansang seed contains some antinutrient compounds like phytate and oxalate (Coulibaly, Adam, et al., 2018; Coulibaly, N'dri, et al., 2018). Their activity lies in their ability to form complexes with metals like Ca. During boiling and roasting, these antinutrients are partially or completely destroyed by high processing temperature. Their destruction is responsible to the increase of Ca concentration in the medium (Makinde & Akinoso, 2013).

As previously observed with the calcium and iron content, the amount of magnesium and phosphorous was significantly higher in processed Djansang seeds compared to the control. The amount of phosphorous obtained in this study was significantly lower than that reported by Ene‐Obong et al. (2018) who obtained a value of 460 mg/100 g with the same seeds in Nigeria. Similar observations were made with magnesium but especially with processed samples. However, the amount of magnesium of the control was significantly lower than that reported by these same authors. The amount of phosphorous and magnesium found in the control and processed Djansang samples in this study were significantly higher than those reported by Coulibaly, Adam, et al. (2018 and Coulibaly, N'dri, et al. (2018 with *Ricinodendron heudelotii* kernel flour. This is the proof that Djansang seeds are good sources of magnesium and phosphorus. Phosphorus together with calcium has been demonstrated as playing a fundamental role in bone mineralization, while magnesium is one of the most important cofactors involved in metabolic reaction (Yokota et al., 2007).

The evolution of potassium and sodium content of Djansang seeds during processing is also presented in Table 3. It is clearly observed that the amount of potassium and calcium has significantly reduced after boiling, compared to roasting. This is the indication that roasting is more suitable for the preservation of sodium and potassium than boiling. This can be explained by the loss of these minerals in the water. The amount of potassium obtained in this study was ranged between 435 and 2,965 mg/100 g. Similar value was reported by Ene‐Obong et al. (2018) (645 mg/100 g). However, the amount of sodium obtained in this study (26.70 to 119 mg/100 g) was significantly lower than that reported by those authors (210 mg/100 g). The fact that the potassium and sodium content significantly decreased during processing has previously be reported by Djikeng et al. (2017) who show that the potassium and sodium content of boiled and roasted walnuts was significantly decreasing than that of the control. The presence of potassium and calcium in Djansang seeds is also beneficial due to their direct relationship with hypertension in humans.

## 4 CONCLUSION

The objective of this study was to evaluate the effect of different processing methods on the physicochemical properties of Djansang. The results of the present investigation showed that the phenolic content and antioxidant activity significantly decreased with boiling. The analysis of the oil quality showed that roasting significantly altered the quality of Djansang oil than boiling. The proximate composition of Djansang seeds (mainly proteins and carbohydrates) was significantly affected by the treatments. However, the effect was more significant with roasting than boiling. All these processing methods significantly increased the amount of phosphorus, iron, calcium, and magnesium. Boiling was preserving better these minerals than roasting. However, the amount of potassium and sodium was significantly lower in boiled Djansang samples than roasted ones. This means that roasting is suitable for the preservation of these nutrients than boiling.

## CONFLICT OF INTEREST

The authors declare that they have no conflict of interest.

## ETHICAL APPROVAL

The study did not involve any human or animal testing.
